# Adverse drug reactions from psychotropic medicines in the paediatric population: analysis of reports to the Danish Medicines Agency over a decade

**DOI:** 10.1186/1756-0500-3-176

**Published:** 2010-06-23

**Authors:** Lise Aagaard, Ebba H Hansen

**Affiliations:** 1 Department of Pharmacology and Pharmacotherapy, Section for Social Pharmacy, Faculty of Pharmaceutical Sciences, University of Copenhagen, Denmark; 2 FKL Research Centre for Quality in Medicine Use, Universitetsparken 2, DK- 2100 Copenhagen, Denmark

## Abstract

**Background:**

The prescribing of psychotropic medicines for the paediatric population is rapidly increasing. In attempts to curb the use of psychotropic medicine in the paediatric population, regulatory authorities have issued various warnings about risks associated with use of these products in childhood. Little evidence has been reported about the adverse drug reactions (ADRs) of these medicines in practice. As spontaneous reports are the main source for information about previously unknown ADRs, we analysed data submitted to a national ADR database. The objective was to characterise ADRs reported for psychotropic medicines in the Danish paediatric population over a decade.

**Findings:**

All spontaneous ADR reports from 1998 to 2007 for children from birth to 17 years of age were included. The unit of analysis was one ADR. We analysed the distribution of ADRs per year, seriousness, age and gender of the child, suspected medicine and type of reported ADR. A total of 429 ADRs were reported for psychotropic medicines and 56% of these were classified as serious. Almost 20% of psychotropic ADRs were reported for children from birth up to 2 years of age and one half of ADRs were reported in adolescents, especially for antidepressants and psychostimulants. Approximately 60% of ADRs were reported for boys. Forty percent of all ADRs were from the category 'nervous and psychiatric disorders'. All but one ADR reported for children below two years were serious and two of these were fatal. A number of serious ADRs reported in children from birth up to 2 years of age were presumably caused by mothers' use of psychotropic medicines during pregnancy.

**Conclusion:**

The high number of serious ADRs reported for psychotropic medicines in the paediatric population should be a concern for health care professionals and physicians. Considering the higher number of birth defects being reported greater care has to be given while prescribing these drugs for pregnant women.

## Background

The prescribing of psychotropic medicines for the paediatric population is rapidly increasing in many countries including Denmark. In attempts to curb the use of psychotropic medicine in the paediatric population, regulatory authorities have issued various warnings about risks associated with use of these products in childhood [1-4]. A systematic review detected seventeen studies since 2000 that reported information about the occurrence of ADRs in paediatric populations [5]. Nearly one third of all ADRs reported in children were due to psychotropic medicines, especially CNS stimulants and antidepressants. However, more detailed information about the characteristics of ADRs from psychotropic medicines in the general paediatric population is lacking and little evidence has been reported about safety and long-term effects of these medicines in practice [6-8]. Lack of knowledge of adverse drug reactions (ADRs) at the point of licensing of new medicines renders spontaneous ADR reporting an important contributor to knowledge about safety of medicines [9]. As spontaneous reports are the main source for information about new and previous unknown ADRs we conducted an analysis of all spontaneous ADR reports for psychotropic medicines in Denmark from 1998 to 2007.

## Methods

We used data from the national Danish ADR database, which contains information about all spontaneous reports submitted to the Danish Medicines Agency (DKMA) [10]. ADRs reported for children from 0 to 17 years of age were included. We analysed the distribution of ADRs per year, seriousness, age and gender of the child, suspected medicine and type of reported ADR (system organ class [SOC]). ADRs were classified as serious on the following criteria: death, life-threatening, requiring hospitalisation or prolongation of existing hospitalisation, resulting in persistent or significant disability/incapacity, a congenital anomaly/birth defect and other medically important conditions.

## Results

### ADRs over time

**Figure 1:**
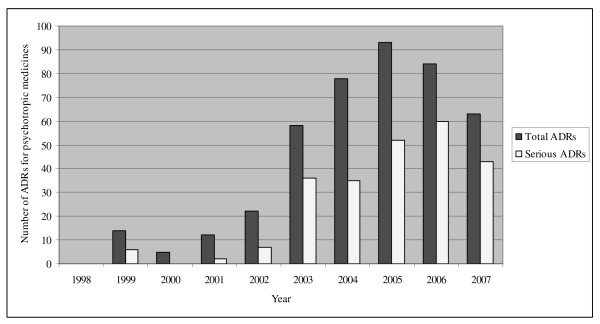
**Annual number of adverse drug reactions (ADRs) for psychotropic medicines reported in the Danish paediatric population**.

From 1998 to 2007 a total of 2437 individual ADR reports containing information about 4500 ADRs were reported for children. Of these, 210 reports corresponding to 429 ADRs were submitted for psychotropic medicines. Figure 1 shows the annual distribution of the reported ADRs. There were wide fluctuations in the number of ADRs reported annually, with an increase in number from 2003 to 2005 followed by a decrease in 2006 and 2007.

### ADRs by age and seriousness

Table 1 shows the distribution of reported ADRs by therapeutic group and medicine, age of patient and seriousness. Almost one fifth of ADRs were reported for children below 2 years and one half of all ADRs were reported for adolescents (from 11 to 17 years of age), and 45% of these were serious. Totally, 59% of all ADRs were reported for boys. More than one half of all ADRs were classified as 'serious'. Table 2 displays characteristics of ADRs reported for children below two years. Two deaths were reported for citalopram and fluoxetin due to chorioamnionitis and persistent foetal circulation, respectively. ADRs among children up to 2 years of age encompassed a wide range of reactions, e.g. convulsion, feeding disorder, neonatal priapism, apnoea and ventricular septal defects. Seven ADRs: drug exposure during pregnancy, neonatal respiratory depression, apnoea and pallor were reported as "maternal drugs affecting foetus". However, the remaining ADRs were probably also caused by mothers' use of psychotropic medicines during pregnancy, as the indications for use were reported as depression, anxiety, panic disorder and schizophrenia. The share of serious psychotropic ADRs was higher than the share of serious ADRs in Danish children in general (40%) [11]. In the general Danish paediatric population, half of all ADRs were reported in children from birth up to 2 years of age, but for psychotropic medicines more than half of all ADRs were reported for adolescents which reflect a more extensive use of psychotropic medicine in 11 to 17- year-olds [9]. The majority of serious ADRs were reported in infants in contrast to reports submitted to Health Canada where 60% of all ADRs for psychotropic medicines were reported in 13 to 19-year-olds, and only 12% in infants [12]. A number of ADRs were reported for Danish children below 2 years, probably due to the mother's intake of psychotropic medicine, primarily antidepressants and antipsychotics, during pregnancy. Serious ADRs such as 'neonatal withdrawal syndrome', 'ventricular septal defects' and 'premature labour' were reported. The risks of malformations as well as preterm delivery of babies due to use of antidepressants during pregnancy have been reported previously in the literature and is supported by our results [13-16].

**Table 1:** Number of adverse drug reactions reported for psychotropic medication in the paediatric population by age and seriousness (in italic) (1998 to 2007)

Age groups (years)	<1	1<2	2-10	11<17	Total
**Antipsychotics (N05A)**					
Levomepromazine	1 *(1)*	0	0	1	2 *(1)*
Zipradison	0	0	2	16 *(11)*	18 *(11)*
Zuclopenthixol	1 *(1)*	0	0	1 *(1)*	2 *(2)*
Chlorprothixene	0	0	0	2	2
Clozapine	0	0	0	1	1
Olanzapine	8 *(8)*	5 *(5)*	0	11 *(3)*	24 *(16)*
Quetiapine	1 *(1)*	0	1 (*1)*	14 *(12)*	16 *(14)*
Sulpiride	0	0	0	3	3
Risperidone	1 *(1)*	0	5	18 *(5)*	24 *(6)*
Aripiprazol	0	0	0	14 *(3)*	14 *(3)*
Total	12 *(12)*	5 (*5)*	8 *(1)*	81 *(35)*	106 *(53)*

**Hypnotics and sedatives (N05B/N05C)**					
Diazepam	1 *(1)*	0	0	0	1 *(1)*
Oxazepam	3 *(3*)	0	0	0	3 *(3)*
Buspirone	1 *(1)*	0	0	0	1 *(1)*
Chloral hydrate	0	0	3 *(3)*	0	3 *(3)*
Midazolam	0	0	1 *(1)*	2 *(2)*	3 *(3)*
Total	5 *(5)*	0	4 *(4)*	2 *(2)*	11 *(11)*

**Antidepressants (N06A)**					
Imipramine	0	0	1 *(1)*	0	1 *(1)*
Clomipramine	1 *(1)*	0	0	0	1 *(1)*
Amitriptyline	2 *(2)*	0	0	0	2 *(2)*
Fluoxetine	14 *(14)*	0	0	1 *(1)*	15 *(15)*
Citalopram	17 *(17)*	4 *(4)*	5 *(2)*	10 *(6)*	36 *(29)*
Paroxetine	5 *(4)*	0	0	6 *(2)*	11 *(6)*
Sertralin	11 *(11*)	0	9 *(3)*	25 *(20)*	45 *(34)*
Escitalopram	1 *(1)*	1 *(1)*	0	0	2 *(2)*
Oxitriptan	0	0	0	3	3
Mirtazapin	1 *(1)*	0	0	12 *(8)*	13 (9)
Venlafaxin	1 *(1)*	0	0	3	4 *(1)*
Total	53 *(52)*	5 *(5)*	15 *(6)*	60 *(37)*	133 *(100)*

**Psychostimulants (N06B)**					
Methylphenidate	0	0	85 *(35)*	44 *(17)*	129 *(52)*
Modafinil	0	0	0	7 *(7)*	7 *(7)*
Atomoxetine	0	0	23 *(7)*	20 *(11)*	43 *(18)*
Total	0	0	108 *(42)*	71 *(35)*	179 *(77)*

**Total N05 and N06**	70 *(69)*	10 *(10)*	135 *(53)*	214 *(109)*	429 *(241)*

**Table 2:** Serious ADRs from psychotropic medicines reported for children below two years of age (1998 to 2007)

ATC	Medicines	Adverse drug reaction	No	Indication of use	Age ofchild
N05A	Levopromazine	Priapism	1	Headache	0
	Olanzapine	Atrial septal defects	1	NA	1
		Blood glucose decreased	1	Schizophrenia	0
		Convulsion	1	NA	0
		Decreased appetite	1	Schizophrenia	0
		Drug exposure during pregnancy	1	NA*	0
		Failure to thrive	1	Schizophrenia	0
		Feeding disorder, neonatal	1	Schizophrenia	0
		Haemoglobin increased	1	NA	1
		Hypoxia	1	NA	0
		Pneumonia	1	NA	1
		Polycythaemia	1	NA	1
		Somnolence	1	Schizophrenia	0
		Tension	1	NA	1
	Risperidon	Drug withdrawal syndrome, neonatal	1	Depression	0
	Sulpirid	Agitation, neonatal	1	Depression	0
	Zuclopemthixol	Supraventricular tachycardia	1	Schizophrenia	0
N05B	Buspirone	Ventricular septal defect	1	Depression	0
	Diazepam	Apnoea	1	Convulsions	0
	Oxazepam	Congenital acrochordon	1	Anxiety depression	0
		Chondropathy	1	Anxiety depression	0
		Drug exposure during pregnancy	1	Anxiety depression	0
N06A	Amitriptyline	Epilepsy	1	Depression	0
		Febrile convulsion	1	Depression	0
	Clompipramine	Psychomotor retardation	1	NA	0
	Citalopram	Tremor, neonatal	3	Panic disorder	1
		Drug withdrawal syndrome, neonatal	2	Depression/Panic disorder	0/1
		Hypotonia, neonatal	2	Depression	0
		Irritability	2	Panic disorder	0/1
		Apnoea	1	NA*	0
		Asthenia	1	NA	0
		Chorioamnionitis	1	Depression	0
		Convulsion, neonatal	1	Depression	0
		Hypertonia	1	Panic disorder	1
		Hypocalcaemia	1	Depression	0
		Neonatal asphyxia	1	Depression	0
		Neonatal respiratory depression	1	NA*	0
		Oral candidiasis	1	Depression	0
		Pallor	1	NA*	0
		Premature labour	1	Depression	0
		Ventricular septal defect	1	Depression	0
	Escitalopram	Atrial septal defect	1	NA	1
		Drug exposure during pregnancy	1	NA*	0
	Fluoxetine	Drug withdrawal syndrome, neonatal	3	Depression	0
		Tremor	2	Depression	0
		Agitation, neonatal	1	Depression	0
		Bradycardia, neonatal	1	Depression	0
		Deafness neurosensory	1	NA	0
		Drug exposure during pregnancy	1	NA*	0
		Dyskinesia, neonatal	1	Depression	0
		Feeding disorder, neonatal	1	Depression	0
		Hypertonia, neonatal	1	Depression	0
		Neonatal disorder	1	Depression	0
		Persistent foetal circulation	1	Depression	0
		Upper limb deformity	1	Depression	0
	Paroxetine	Klinefelter's syndrome	1	NA	0
		Neonatal respiratory failure	1	NA*	0
		Psychomotor retardation	1	NA	0
	Sertraline	Respiration abnormal	2	Depression	0
		Cerebral palsy	1	Depression	0
		Circulatory collapse	1	Depression	0
		Cyanosis	1	Depression	0
		Drug withdrawal syndrome, neonatal	1	Depression	0
		Drug exposure during pregnancy	1	Depression	0
		Muscle spasms	1	Anxiety	0
		Myoclonus	1	Anxiety	0
		Persistent foetal circulation	1	Depression	0
		Pulmonary hypertension	1	Depression	0
	Mirtazapine	Cerebral palsy	1	Depression	0
	Venlafaxine	Agitation, neonatal	1	Depression	0

*Indication for use not listed, but recorded as "Maternal drugs affecting foetus"; NA: not available

### ADRs by therapeutic subgroups

The largest share of ADRs (42%) was reported for psychostimulants (ATC group N06B), followed by 31% for antidepressants (ATC group N06A) and 24% for antipsychotics (ATC group N05A). More than one half of the ADRs reported for antipsychotics were caused by the drugs ziprasidone, olanzapine and risperidone. Although only 2.5% of ADRs were reported for anxiolytics and sedatives (ATC group N05B and N05C), predominantly in infants, all these ADRs were serious. Two-thirds of the ADRs reported for antidepressants (ATC group N06A) were reported for infants and adolescents and exclusively for the medicines sertraline, citalopram and fluoxetine and seventy-five percent of these were serious. For psychostimulants (ATC group N06B) 50% of ADRs were serious and reported for children from six to nine years of age, and 40% of the reports were associated with methylphenidate and atomoxetine. With one exception, all 70 ADRs reported for children less than one year of age were serious.

### ADRs by type

Table 3 shows the distribution of reported ADRs by system organ class (SOC). The largest shares of ADRs were reported for the SOCs 'psychiatric disorders' (20% of total), 'nervous system disorders' (20% of total) and 'general disorders and administration site conditions' (12% of total). Less than 1% of the total number of reports concerned the following SOCs: 'ear and labyrinth disorders', 'endocrine disorders', 'hepatobiliary disorders', 'immune system disorders', 'pregnancy, puerperium and perinatal conditions' and 'surgical and medical procedures'. The largest shares of serious ADRs, about 25% of all were reported for the SOCs: 'psychiatric disorders' and 'nervous system disorders'. The distribution between serious and non-serious ADRs within SOCs varied. More than seventy-five percent of ADRs reported from the SOCs: 'nervous system disorders', 'musculoskeletal and connective tissue disorders' and 'congenital, familial and genetic disorders'were serious.

**Table 3:** Adverse drug reactions from psychotropic medicines by system organ class (descending order)

System Organ Class (SOC)	All ADRs (%)	Serious as % of all ADRs	Serious as % of ADRs
	N = 429	N = 241	
Psychiatric disorders	20	25	70
Nervous system disorders	19	26	77
General disorders and administration site conditions	12	7	33
Skin and subcutaneous tissue disorders	8	3	21
Gastrointestinal disorders	8	6	41
Cardiac disorders	5	6	67
Investigations	5	6	67
Respiratory, thoracic and mediastinal breast disorders	4	4	59
Metabolism and nutrition disorders	3	2	38
Musculoskeletal and connective tissue disorders	3	4	77
Congenital, familial and genetic disorders	2	4	77
Reproductive system and breast disorders	2	1	22
Vascular disorders	2	2	56
Others	7	4	33

### Implications

There are huge gaps in the evidence on the safety of medicines in children as only few medicines prescribed for children are tested in clinical trials and licensed for use in this population. In addition, information about serious and long-term ADRs is sparse due to the limitations embedded in the design of randomised, controlled clinical trials which are used primarily to test hypotheses about efficacy rather than safety and children are usually excluded from clinical trials of medicines for ethical reasons [17]. Therefore, it is very important to systematically analyse and evaluate data reported to the spontaneous reporting programmes as these reports are the major source for new information about possibly serious and previously unknown ADRs [18]. Several of the reported ADRs were birth defects, an area where we have very limited knowledge.

## Conclusions

The high number of serious ADRs reported for psychotropic medicines in the paediatric population should be a concern for health care professionals and physicians. Considering the higher number of birth defects being reported greater care has to be given while prescribing these drugs for pregnant women.

## Competing interests

The authors declare that they have no competing interests.

## Authors' contributions

LA and EHH designed the study, analysed data and wrote the first version of the manuscript. LA did the sampling. Both authors read and approved the final version of the manuscript.
